# The Role of *Danio rerio* in Understanding Pollutant-Induced Gut Microbiome Dysbiosis in Aquatic Ecosystems

**DOI:** 10.3390/toxics13090769

**Published:** 2025-09-11

**Authors:** Svetlana G. Frolova, Aleksey A. Vatlin, Iunona Pospelova, Nikita A. Mitkin, Gulnara A. Kulieva, Vsevolod V. Pavshintsev

**Affiliations:** 1 Department of Biosciences, University of Milan, 20122 Milan, Italy; 2 Institute of Environmental Engineering, RUDN University, 6 Miklukho-Maklaya Street, 117198 Moscow, Russia; 3 Quantori, Cambridge, MA 02142, USA

**Keywords:** zebrafish, microbiome, pollutants, heavy metals, pesticides, microplastic, antibiotics

## Abstract

Freshwater pollution is a global issue that can impact aquatic organisms in multiple ways. One of the many detrimental consequences of freshwater pollution is the disruption of the intestinal microbiome in aquatic animals. This review addresses the impact of various chemical entities like pesticides, heavy metals, antibiotics, dyes, and microplastic. Gut microbiota serves as a crucial regulator of metabolic processes across all organisms. Since numerous metabolic pathways are coordinated by microbial communities, even minor disruptions can lead to consequences ranging from mild to severe. The widespread use of chemicals in modern life has made them a primary focus of current gut microbiota research. Zebrafish (*Danio rerio*) can serve as a model organism to investigate gut microbiome responses to exposure to hazardous contaminants. In this review we include research studying pesticides (methomyl, λ-cyhalothrin, cyproconazole, dieldrin, penthiopyrad, acetochlor, metamifop, imidacloprid, difenoconazole, imazalil, cypermethrin), heavy metals (lead, cadmium, arsenic, chromium, copper, and various nanoparticles), antibiotics (oxytetracycline, florfenicol, doxycycline, trimethoprim, erythromycin, streptomycin, tetracycline, sulfamethoxazole, and clarithromycin), and microplastics (polystyrene, polyethylene, polyester, polypropylene). This review study provides a description of microbiome alterations due to single and combined short- and long-term exposure to the aforementioned contaminants in zebrafish and larvae microbiomes.

## 1. Introduction

The varied microbial populations inhabiting the gut, collectively called the gut microbiota, are essential for regulating metabolism, modulating the immune system, producing fatty acids and vitamins, preserving intestinal balance, and influencing brain health and behavior [1,2,3,4]. The microbiome is a dynamic system that can be affected by a combination of multiple factors including genotype, different types of chemotherapy, diet composition, lifestyle, social interactions, and environmental exposure to various xenobiotics. Changes in microbiota composition or function, known as dysbiosis, are linked to a range of common metabolic and immune disorders, including obesity, diabetes [5], hepatic diseases [6], Crohn’s disease [7], inflammatory bowel disease [8], colorectal cancer [9], and allergy [10]. Furthermore, there is evidence of the bidirectional communication between the gut microbiome and the central nervous system, referred to as the “microbiota–gut–brain axis” [4,11]. Moreover, dysbiosis was marked as one of the 12 newly acknowledged hallmarks of aging, further underscoring the significance of the microbiome [12]. Playing important roles in various biological processes, the gut microbiota is highly intricate and fragile, so any disruption may lead to biological changes. As a result, negative impacts on the gut microbiota can be significant, ultimately influencing the health of organisms.

The extensive use of synthetic chemicals in various industrial and agricultural sectors has resulted in widespread environmental contamination, raising global concerns about their impact on human and animal health. Agricultural, urban, mining, pharmaceutical manufacturing, and other industrial activities have each played a role in increasing the concentrations of heavy metals, pesticides and herbicides, dyes, antibiotics, and other medicines in aquatic systems. Pollution may negatively influence aquatic life by lowering dissolved oxygen concentrations, modifying water pH, and introducing toxic substances into the aquatic environment. These contaminants can accumulate in organisms, seriously harming the ecological environment, aquatic habitats, and human health. To elucidate the effects of freshwater pollution on aquatic vertebrates with translational relevance to mammalian systems, it is valuable to investigate single-contaminant exposures using appropriate model organisms, such as fish models that are directly susceptible to waterborne contaminants. Freshwater contaminants have harmful effects on fishes, leading to histopathological changes in gills, kidneys, and the liver [13]; DNA damage [14]; a decrease in growth rate and in red blood cells [15]; inflammation [16]; and neurotoxicity [17]. There are accumulating numbers of studies demonstrating how freshwater contaminants lead to alteration of the microbiome in aquatic habitats such as amphibians [18,19] and fishes [20,21,22,23]. It is well established that the gut microbiome is highly sensitive to environmental xenobiotics, including drugs, dietary components, and pollutants. Moreover, the gut microbiome plays a pivotal role in determining the fate of these xenobiotics by modulating host xenobiotic metabolism and acting as a barrier to prevent systemic toxin absorption [24]. The gut microbial community in fish plays key roles in essential biological processes, including nutrition, physiology, and immunology, which contribute to maintaining their overall health [25]. From an ecotoxicological perspective, the gut microbiota of fish has received limited attention as a potential indicator for evaluating environmental quality. Understanding its composition could provide insights into both the health of the host and the condition of the surrounding environment. Research using zebrafish offers a valuable platform to investigate how gut microbial imbalances affect host health and to explore potential strategies for restoring microbial homeostasis.

The main techniques to investigate the effects of chemical substances on the gut microbiota of zebrafish are high-throughput sequencing, metagenomics, metabolomics, metabolic pathway analysis, gene expression, and molecular analysis. This review examines the impacts of pesticides, antibiotics, metal nanoparticles, and microplastics on the gut microbiome of zebrafish, with a focus on the associated metabolic pathways, to provide deeper insights into how water contaminants affect fish health.

## 2. Zebrafish and Gut Microbiome

### 2.1. Danio rerio as a Model for Gut Microbiota Study

The popularity of zebrafish in life science research stems from their compact size, prolific breeding, transparent embryos, fast external development, straightforward laboratory maintenance, and extensive genomic data. Large numbers of zebrafish can be maintained in a shared, easily sampled aquatic environment, enabling extensive biological replication. Comparison with the human reference genome shows that approximately 70% of human genes have at least one obvious zebrafish orthologue [26]. Additionally, given that gut functions and immune genes are relatively conserved between zebrafish and mammals, findings from zebrafish studies can often be translated to mammalian systems [27]. Zebrafish are an attractive model for gut microbiota research due to their many advantageous characteristics. The intestines of zebrafish and mammals are highly homologous in terms of developmental processes, structural composition, and biological function. Furthermore, the expression patterns of antimicrobial genes and the distribution of leukocytes along the anterior–posterior axis of the intestine are relatively conserved between zebrafish and mammals [28].

Zebrafish and mammals have similar gut microbial compositions, with six shared bacterial phyla: Proteobacteria, Fusobacteria, Firmicutes, Bacteroidetes, *Actinobacteria*, and Verrucomicrobia [29]. However, while the human gastrointestinal tract is predominantly composed of Firmicutes, Bacteroidetes, and *Actinobacteria* at the phylum level, the zebrafish gut is mainly dominated by Proteobacteria—particularly the genera *Aeromonas*, *Pseudomonas*, and Vibrio—followed by Firmicutes or Fusobacteria [30]. Although the dominant phyla in zebrafish differ from those in humans, the gut microbiota of zebrafish triggers relatively conserved host responses during colonization and development [31]. Another advantage of using zebrafish in microbiome research is that it is relatively easy to establish germ-free (GF) zebrafish larvae to investigate the functions of particular intestinal microorganisms by introducing them in larvae [32].

In summary, zebrafish represent a unique model organism for studying gut microbiome dynamics. Their genetic similarity to humans, conserved intestinal and immune functions, and ease of maintenance make them an invaluable tool for investigating host–microbiota interactions and the effects of environmental influences on gut health.

### 2.2. Pesticides and Gut Microbiota

Pesticides are hazardous chemical substances or mixtures used by humans to enhance crop yields and protect them from pests, as well as to control insects that spread diseases. Generally, pesticides are classified into four types: insecticides, fungicides, herbicides, and bactericides. These contaminants can enter the aquatic ecosystem through runoff from agricultural fields, urban areas, and industrial wastewater. In urban and rural water, pesticides have been identified at high concentrations across the world [33,34,35]. Pesticides pose significant risks to human health, with exposure linked to various conditions including cancer, respiratory diseases such as asthma, neurodegenerative disorders like Parkinson’s disease, psychological effects such as anxiety, as well as skin allergies, eye irritation, and severe fatigue [36]. The entry of pesticides into the intestines can cause dysbiosis of gut microbiota [37]. Pesticides are highly toxic to aquatic organisms such as snails, fish, etc. Furthermore, some pesticides are ecologically persistent and can remain in the aquatic system for extended periods. As such, it is essential to monitor and reduce pesticide usage to minimize their impact on aquatic ecosystems [38]. With their toxic effect, pesticides can cause severe biochemical and histological changes as well as neurological dysfunction in freshwater organisms [39,40]. All presented studies on pesticides and the zebrafish microbiome are summarized in Table 1.

**Methomyl** is an oxime carbamate pesticide applied to control a wide range of arthropods which has a high solubility in water and long half-life [53]. Methomyl can easily contaminate surface and groundwater, which poses a potential threat to the non-target environment and human safety. Exposure of juvenile zebrafish to methomyl at an environmentally relevant concentration for 56 days resulted in damage to intestinal villi and significant shifts in the abundance of various bacterial genera, including *Shewanella* and *Pesudomonas*, which serve as probiotics capable of improving growth and resistance to pathogens in juvenile fish. Methomyl exposure disrupts the zebrafish gut microbiome by interfering with the tricarboxylic acid (TCA) cycle, which is positively correlated with altered bacterial composition (particularly *Shewanella*, *Rubrobacter*, *Pseudomonas*, and *Acinetobacter*), while suppressing amino acid metabolism and fatty acid degradation even at low concentrations (0.05 mg/L). *Shewanella* and *Pseudomonas* also exhibiting strong correlations with the transcription levels of inflammation-related genes [41].

**λ-cyhalothrin** (LTC) is a broad-spectrum synthetic type II pyrethroid insecticide used to control pests in various crops. After 21-day LTC exposure at an environmental concentration, the adult zebrafish gut microbiota showed an altered composition with decreased beneficial Firmicutes (which support energy absorption and anti-inflammatory effects) and significant changes in Fusobacteriota and Proteobacteria levels (linked to inflammation and immune responses), resulting in increased oxidative stress and compromised gut immune function [42].

**Cyproconazole** (CPZ), one of the most popular triazole fungicides with a half-life of >40 days in water, is widely used for its excellent bacteriostatic effect. In adult zebrafish, exposure to an environmentally relevant concentration of CPZ after 40 days caused changes at the phylum level: Proteobacteria, Bacteroidetes, Firmicutes, and Verrucomicrobia were reduced considerably, while Fusobacteria and Tenericutes were significantly increased. An increase in *Desulfovibrio* genus bacteria in the gut, which produce excess H_2_S (accounting for 60% of gut H_2_S) [54], contributed to intestinal mucin degeneration (this result aligns with a decrease in goblet cells producing mucin) and inflammation associated with inflammatory bowel disease, while beneficial Verrucomicrobia (which produce important short-chain fatty acids for gut health and immune function) typically abundant in healthy individuals were reduced [43].

Dietary exposure of adult zebrafish to environmentally relevant doses of **dieldrin**, an organochlorine pesticide, showed that a dose of 16 ng/g induced changes in the abundance of Firmicutes: it decreased the Firmicutes phylum (particularly *Clostridia*, which represents 95% of Firmicutes) and *Betaproteobacteria* and increased Verrucomicrobia species. A computational analysis predicted that the compound caused alterations in metabolic pathways involved in bacterial heme biosynthesis, selenium metabolism, energy metabolism (affected acetyl-CoA pathway intermediates), and bacterial cell wall peptidoglycan synthesis [44]. These pathways of selenium and heme metabolism are crucial for many health aspects and their dysregulation is a marker of diseases; for example, selenoproteins play essential roles in protecting specific cells, such as neurons, from oxidative damage [55].

**Penthiopyrad** (PTH) is a fungicide, which is used against various plant diseases including rust, gray mold, apple scab, wheat disease, etc. [56]. In the PTH treatment group of adult zebrafish, there was a significant increase in Firmicutes and in Proteobacteria and a decrease in Fusobacteria. The increase in Proteobacteria might indicate intestinal inflammation, as this phylum’s abundance is associated with inflammatory processes and oxidative stress. The reduction in Fusobacteria suggests a potential vitamin deficiency, since genera like *Cetobacterium* within this phylum are important for vitamin synthesis [45].

Two microbiomics studies were conducted in adult zebrafish exposed to **imidacloprid** (IMI), an insecticide primarily used to control insect infestations. Luo et al. found that exposure to low environmentally relevant concentrations of IMI caused limit alterations in the microbiome; significant changes were only found in the increase in *Fusobacteria* and *Bacteroides* and the decrease in Proteobacteria and Verrucomicrobita. The relative abundances of *Flavobacterium*, *Flavobacteriaceae*, *Neisseriiaceae*, and *Acetobacteraceae* were increased and might explain the reason for the increase in Lipopolysaccharides and in the inflammation level in the gut of zebrafish [49,57]. Huang et al. studied the effect of IMI on adult zebrafish in presence and absence of light. IMI exposure significantly altered gut microbial diversity, reduced Proteobacteria, increased Fusobacteria, and shifted microbial composition at both the phylum and genus levels. Interestingly, extended darkness minimized IMI-induced disruptions, preserving microbial diversity, composition, and predicted metabolic functions [47].

The effect of **acetochlor**, a widely used herbicide, leading to an increase in Proteobacteria could indicate a disruption of gut health, as proteobacterial expansion is linked to increased intestinal permeability in adult zebrafish and may contribute to metabolic disorders and inflammatory bowel disease [46].

Adult zebrafish exposed to sublethal concentrations of **metamifop**, a novel herbicide that mainly causes lipid synthesis disorder and leads to the growth retardation of weeds, experienced inflammation, decreased goblet cell numbers, and impaired lipid absorption. Significant changes in the microbiota were identified only at the family and genus levels: decreased genera *Psychrobacter* and *Aeromonas* and increased *genera Rhodobacter*, *Pelomonas*, and *Ralstonia*. *Psychrobacter* and *Aeromonas* are both beneficial bacteria that play crucial roles in the fish intestine. *Psychrobacter* enhances nutrient utilization and innate immunity while contributing positively to microbial diversity [58]. Similarly, *Aeromonas* secretes digestive enzymes and helps regulate the immune system, supporting overall gut health [59]. On the other hand, *Ralstonia* is identified as a pathogenic bacteria [48].

In adult zebrafish, exposure to **difenoconazole** at concentrations resulting in acute toxicity led to an increased abundance of Firmicutes, Aeromonas, Enterobacteriaceae, and Bacteroides. Microbiomic analysis further revealed that gut microbiota may play a role in lipid metabolism and liver toxicity during zebrafish development [50].

The fungicide **imazalil** (IMZ) at environmentally relevant concentrations at the phylum level changed the composition of the gut microbiota of adult zebrafish. Proteobacteria and Bacteroidetes decreased, while Fusobacteria and Firmicutes increased. Specifically, the abundances of beneficial bacteria such as *Bacteroides*, *Alistipes*, and *Akkermansia*, which are involved in mucin degradation and bile acid metabolism, were significantly reduced. Notably, Bacteroides suppressed secondary bile acid production, and *Rhodobacter*, linked to secondary bile acid synthesis, was reduced nearly tenfold. Conversely, the abundances of potentially pathogenic bacteria like *Stenotrophomonas* and *Mycoplasma* increased [51].

Fang et al. studied the co-exposure of polystyrene microplastics (PS-MPs) and **cypermethrin** (CYP) at low- and high-environmentally relevant concentrations in adult zebrafish for 21 days. The synergistic effect of these two pollutants worsened dysbiosis of the gut microbiota and metabolic dysfunction. PS-MPs might act as carriers for CYP, increasing intestinal accumulation of CYP via surface adsorption, thereby amplifying CYP’s negative effects. A significant reduction in beneficial bacterial taxa such as Fusobacteriota and Firmicutes and the promotion of pathogenic genera, including *Preplasmiviricota*, with higher abundances were observed in combined exposure. Additionally, CYP and co-exposure induced a more complex ARG–microbe interaction network than PS-MPs alone [52].

### 2.3. Medicines and Gut Microbiota

#### 2.3.1. Antibiotics

The presence of antibiotic residues in environmental systems has emerged as a major ecological concern in recent years. The extensive application of antimicrobial agents across medical treatment, farming practices, and fish cultivation has resulted in the discharge of these bioactive substances into aquatic environments [60,61]. Antibiotics enter freshwater systems via wastewater discharge, agricultural runoff, and improper pharmaceutical disposal, where they disrupt microbial communities by promoting antibiotic-resistant bacteria (ARB) and the spread of antibiotic-resistance genes (ARGs). Revised studies related to antibiotics and exposure to other medicines are presented in Table 2.

Due to its high activity and low toxicity, **florfenicol** (FFC) is one of the most widely used antibiotics in livestock, poultry, and aquaculture [76]. However, FFC can accumulate in water, reaching concentrations ng/L∼μg/L [77]. FFC itself or its metabolites can accumulate in the environment to certain levels, potentially impacting both target and non-target microbial communities and posing risks to human health via transfer through the food chain.

Adult zebrafish were exposed to different concentrations of FFC, including 5 mg/L, 20 mg/L (T20), and 40 mg/L (T40). FFC exposure has a complex influence on the fish gut microbiota. Across all tested concentrations, FFC reduces both microbial diversity and evenness within the intestinal community. Notably, concentrations above 5 mg/L significantly suppress the abundance of opportunistic pathogens like *Vibrio*, *Mycobacterium*, *Flavobacterium*, and *Aeromonas*, which allows beneficial bacteria such as rhizobial groups to proliferate, potentially improving the overall gut environment. The most significant effects on the gut microbiota were observed at the higher concentrations (T20 and T40 groups) with reduction in the phyla containing many conditional pathogenic Gram-negative bacteria, especially the phyla Proteobacteria and *Actinobacteria*. However, FFC at 5 mg/L or higher also triggers increased expression of ATP-binding cassette transporters in gut microbes, which can enhance the mobility of antibiotic resistance genes and facilitate their spread. Additionally, FFC-induced shifts activate metabolic pathways that consume more host energy, which may negatively affect fish growth and development. Overall, while FFC can help control harmful bacteria, its use also carries risks for resistance dissemination and host health [64].

Consistent with earlier findings, exposure of adult zebrafish to FFC resulted in a pronounced increase in Proteobacteria abundance within the gut microbiota, accompanied by a reduction in Fusobacteria populations. Notably, this dysbiosis persisted even after discontinuation of FFC treatment. Following challenge with *Aeromonas hydrophila*, zebrafish that had received FFC-medicated feed exhibited significantly higher mortality rates than those fed a standard diet, suggesting a connection between antibiotic-induced microbiome disruption and increased disease susceptibility [63].

In the study by Yang et al., adult male zebrafish were exposed to three different veterinary antibiotics, **doxycycline** (DH) and **oxytetracycline** (OTC), and FFC at different concentrations [62]. The maximum detected concentration of OTC in freshwater aquaculture systems was reported to be 7028 ng/L [78]. At the phylum level, the relative content of Proteobacteria increased in the OTC and FF groups with concentrations of 100 μg/L. The relative content of Fusobacteria also decreased after DH, OTC, or FF exposure. The microbiome changes suggest that DH, FF, and OTC exposures may disrupt gut health by reducing beneficial bacteria such as *Pediococcus*, promoting pathogenic or inflammation-associated genera (e.g., *Ralstonia*, *Escherichia shigella*, *Dorea*) and altering microbial balance in ways seen in obesity, liver disease, and neurological disorders [67].

Another study investigated the alteration of the gut microbiota in zebrafish in chronic exposure to OTC, **sulfamethoxazole**/**trimethoprim** (SMZ/TMP), or **erythromycin** (ERY). Chronic exposure to these antibiotics changed the gut microbiome composition in zebrafish for two of the three antibiotics tested. In zebrafish treated with SMZ/TMP, there were significant changes in the abundance of Fusobacteria and Proteobacteria at the phylum level, and in *C. somerae* and *Cellvibrio fibrivorans* at the species level. In zebrafish treated with ERY, significant changes were seen in Proteobacteria and Firmicutes at the phylum level, and *C. somerae*, *Aeromoas veronii*, and *KM585593* at the species level. However, no significant changes were observed in the microbiome of fish treated with OTC, likely because tetracyclines degrade quickly when exposed to light. After 30 days all three treated groups were challenged with *Edwardsiella piscicida* (*E. piscida*), a Gram-negative fish pathogen. Intriguingly, despite clear evidence of gut microbiome dysbiosis across all antibiotic treatments, the susceptibility to *E. piscicida* infection decreased in antibiotic-treated groups compared to controls. These findings highlight that, contrary to expectations based on gut dysbiosis, chronic antibiotic-induced alteration of the gut microbiome did not correspond to increased vulnerability but instead was associated with decreased susceptibility to *E. piscicida* infection in zebrafish [62].

Another study focused on the combined effects of micro-/nanoplastics and OTC in adult zebrafish identified that OTC exposure significantly decreased the relative abundance of Bacteroidetes compared to the control group, consistent with previous findings. Conversely, the abundance of Fusobacteriota increased, which is possibly a response to severe intestinal damage caused by OTC, as some members of Fusobacteriota metabolize carbohydrates and mucins into butyrate, an SCFA involved in defending against inflammation and carcinogens [65].

Qiu et al. investigated the immunosuppressive effects of antibiotic **enrofloxacin** (ENR) at an environmentally relevant concentration range 0.01–100 μg/L and its potential associations with the intestinal microbiota of zebrafish larvae. They found a total of 22 bacterial taxa were significantly changed and correlated with markers of immunosuppression. These taxa, including *Rickettsiales*, *Pseudomonadales*, and *Flavobacteriales*, also considered to be common pathogens, were found to be correlated with immunosuppressive biomarkers, such as a decrease in the abundance of macrophages and neutrophils. Interestingly, the proportion of *Flavobacteriales* was increased in the ENR-treated zebrafish gut together with one-third of fluoroquinolone resistance genes in the zebrafish gut. This suggests that *Flavobacteriales* is a class of antibiotic-resistant bacteria [66].

Another study investigated the effect of exposure of two different environmental concentrations of OTC and SMZ in a period from larval stage to adulthood (120 days). SMZ has been found to exceed a concentration of 100 ng/L in surface water [79]. In this study, antibiotic-exposed samples showed significantly higher abundances of *Flavobacterium* species compared to controls. While some *Flavobacterium* species are part of the healthy fish gut microbiome, they are also known pathogens causing diseases such as cold-water disease, rainbow trout fry syndrome, columnaris disease, and bacterial gill disease. The ARGs sul2 and tet(X6) were significantly more abundant in OTC-exposed samples, with sul2 positively correlated with SMZ concentration, suggesting that even low environmental antibiotic concentrations can increase ARG abundance. Both tet(X6) and sul2 correlated strongly with *Flavobacterium* abundance in antibiotic-exposed samples, implying possible co-occurrence and linked resistance [68].

Almedia et al. studied alterations in the water and the gut microbiome of adult zebrafish after 2 months of exposure to low (10 g/L) and high (10,000 μg/L) concentrations of OTC. Indeed, in water samples, effects were observed even in the lowest concentration tested with the increase in the *Deltaproteobacteria* class, while in the zebrafish gut, effects were only observed in the highest concentration. The families *Alphaproteobacteria* and *Rhodobacteraceae*, linked to fish health and probiotic properties (e.g., producing tropodithietic acid-inhibiting pathogens), were increased. There was an increase in *Gordonia genus* (*Actinobacteria*), which is important for the biodegradation of pollutants but also includes human pathogens with infection potential, suggesting both beneficial and risk factors from its increased abundance. A decline in *Gammaproteobacteria*, which includes many fish pathogens like *Shewanella*, was observed, possibly due to OTC being an antibacterial spectrum affecting pathogenic bacteria prevalent in the fish gut [69].

Zebrafish larvae were exposed to subclinical concentrations of **streptomycin**; exposure caused a shift from mixed communities of Proteobacteria and Bacteroidetes to communities dominated predominantly by Proteobacteria. The most abundant *Proteobacteria* genus after streptomycin exposure was *Sphingomonas*, which is not normally associated with healthy zebrafish microbiomes. *Sphingomonas* includes species known as human pathogens and that harbor resistance mechanisms against streptomycin [70].

Tetracycline (TET), widely used across human, veterinary, and aquaculture applications, is the most commonly detected antibiotic in aquatic environments with concentrations reaching 158 μg/L [80]. Antibiotic exposure can affect body weight and lead to obesity. In the study by Keerthisinghe et al., the effect of **tetracycline** (TET) on the body weight of juvenile zebrafish was explored. In concentrations of 1 and 100 µg/L, it was shown that TET increased the zebrafish body weight with hepatic lipid accumulation. Exposure to 1 µg/L TET elevated the ratio between Bacteroidetes and Firmicutes to 2.9, whereas this ratio was markedly decreased to 0.19 under 100 µg/L TET [71]. Previously, this ratio has been described as being higher in obese mice than in normal-weight mice [81]. In addition, an increase in the abundance of Firmicutes is usually considered as an obesogenic factor [82]. Also, the class of *Ersipelotrichia* was enriched in the 1 μg/L TET group; previously, it has been reported to be increased in obese animals due to their high-fat diet [83]. That potentially explains the higher weight gain observed at the lower concentration [71].

Exposure of zebrafish larvae to environmentally relevant concentrations (0.01 mg/L each) of the antibiotics SMZ and **clarithromycin** (CLA) for two weeks resulted in significant dysbiosis of the intestinal microbiota. Specifically, there was an increased relative abundance of *Chloroflexia* and *Alphaproteobacteria*, concurrent with reductions in *Actinomycetia*, *Bacilli*, and *Gammaproteobacteria*. Fungal communities (mycobiota) were also affected, with increased total fungal abundance: *Sordariomycetes* significantly increased following antibiotic exposure, while classes such as *Saccharomycetes*, *Dothideomycetes*, and others decreased in their relative abundance. The SMX+CLA-exposed group was more susceptible to spring viremia of carp virus (SVCV). Even a sublethal dose of SVCV induced a certain mortality in the antibiotic-treated fish. The dysbiotic microbiota, alongside a reduced number of specific immune cells, likely contributed to higher susceptibility to viral pathogens [72].

#### 2.3.2. Hormones and Antidepressants

Other medical compounds such as antidepressants and hormones can also be identified in freshwater bodies. **Amitriptyline** (AMI) is the most commonly prescribed tricyclic antidepressant, and its’ metabolites have been detected at 0.34–6.71 μg/L in the influent samples in wastewater treatment plants (WWTPs) [84] and at 7.46 μg/L in contaminated groundwater [85]. Short-term (7-day) exposure of adult zebrafish to AMI caused a reduction in beneficial, metabolically active gut bacteria and increased opportunistic pathogens in adult zebrafish. There was a decline in beneficial bacterial genera involved in metabolic processes, such as *Exiguobacterium* and *Methylobacterium* (linked to carbon metabolism), and an increase in genera capable of degrading environmental pollutants (e.g., *Rhodococcus* and *Acidovorax*), likely due to structural similarities between AMI and polycyclic aromatic hydrocarbons. During the 21-day recovery period in AMI-free water, gut damage did not resolve and, in fact, worsened, with an increased number of mucus-secreting goblet cells and further reductions in beneficial and metabolically active bacterial genera (such as *Paracoccus* and *Hyphomicrobium*, involved in nitrogen cycling) [73].

**Cortisone**, a crucial natural glucocorticoid widely used to treat various diseases, has been detected in WWTPs at 45.0 ng/L and at 0.1–433.0 ng/L in rivers [86,87]. Chronic exposure to cortisone at environmentally relevant concentrations significantly disrupted the composition and function of the adult zebrafish gut microbiota, such as through an increase in the abundance of Proteobacteria and a shift in the Firmicutes-to-Bacteroidetes ratio. Fusobacteria, especially the genus *Cetobacterium*, known for their roles in glucose metabolism, intestinal health, and antiviral immunity, increase in response, potentially helping to restore intestinal balance [74].

**Norethindrone** (NET), a synthetic progestin prescribed in human hormone therapy and contraceptives and in veterinary medicine, can be absorbed by microplastics in water environments [75]. After 30 days of co-exposure of adult zebrafish to NET and polystyrene microplastics (PS) the gut microbiota alterations exhibited some degree of sexual dimorphism, with dominant phyla differing between females and males: female zebrafish guts were mainly dominated by Bacteroidetes, Proteobacteria, and Firmicutes, while male zebrafish guts were dominated by Fusobacteria and Proteobacteria. The genus *Cetobacterium* decreased significantly in the combined PS+NET exposure group compared to both single exposure groups. *Aeromonas*, a known fish pathogen implicated in conditions such as enteritis and sepsis, showed increased abundance under co-exposure [75].

### 2.4. Heavy Metals

The term “heavy metals and metalloids” refers to elements possessing atomic densities above 4 g/cm^3^, which comprise copper (Cu), cadmium (Cd), zinc (Zn), lead (Pb), mercury (Hg), arsenic (As), silver (Ag), chromium (Cr), iron (Fe), and platinum (Pt) [88]. These metals reach marine ecosystems via natural processes, including volcanic activity, hydrothermal vent emissions, and rock weathering, as well as through anthropogenic inputs, such as industrial emissions, metal smelting, petrochemical production, mining, and wastewater discharge release, which often introduce them at rates far exceeding those from natural sources [89]. Mercury, cadmium, and lead are highly toxic and resistant to degradation, enabling their persistence in aquatic environments long after their release [90]. Once environmental heavy metal levels become elevated, aquatic environments serve as repositories that concentrate these contaminants to greater degrees. Metal exposure in aquatic organisms occurs through two primary pathways: direct uptake from abiotic compartments including water and sediment, and indirect acquisition via consumption of contaminated prey and food sources. Together, these pathways drive the accumulation of heavy metals within food webs, resulting in progressively higher concentrations at successive trophic levels [91]. Gills serve as a primary site for the uptake of waterborne metals, including copper (Cu) and zinc (Zn), a process that is particularly pronounced in early life stages or in species with elevated metabolic and ventilation rates [92]. Predatory species at higher trophic levels generally show increased mercury bioaccumulation via biomagnification, while deposit-feeding organisms ingest sediment-bound metals that display poor trophic transfer efficiency but accumulate in benthic habitats [93].

All studies related to heavy metal and metal nanoparticle exposure are summarized in Table 3.

#### 2.4.1. Lead (Pb)

Xia et al. explored the dynamic effects of environmentally relevant concentrations of lead (Pb) exposure on the gut microbiota and neurobehavior of zebrafish larvae during critical early developmental stages (5 to 7 days post-fertilization). They revealed significant, time-dependent shifts in microbial community composition alongside concurrent changes in locomotor activity, implicating gut microbiota dysbiosis in Pb-induced neurotoxicity. In the Pb-exposed group of larvae, with increasing Proteobacteria and decreasing *Actinobacteria* over time, seven genera showed significant correlations with locomotor activity indicators, including *Rhodococcus*, *Deinococcus*, *Bacillus*, *Bosea*, *Bradyrhizobium*, *Staphylococcus*, and *Rhizobium*. *Rhizobium* abundance increased over time in Pb-exposed larvae and correlated negatively with neurobehavior performance. It is associated with intestinal barrier damage and inflammation, suggesting a role in neurotoxicity [94]. Other genera like *Rhodococcus* and *Bosea* have been implicated in neurodevelopment and psychological health in humans and model organisms, linking microbiota shifts to behavioral outcomes [108,109].

Short-term exposure to environmentally relevant concentrations of Pb (10 and 30 μg/L) on male adult zebrafish notably affected bacterial genera including *Bacteroides*, *Flavobacterium*, *Ralstonia*, *Alloprevotella*, *Roseburia*, and *Ruminococcus*, which are closely linked to host metabolism, disease resistance, and inflammatory responses. Some genera such as *Alloprevotella* have been associated with reduced cardiovascular disease risk and immune modulation, underscoring the functional relevance of these microbial shifts [96,110].

Wang et al. investigated the synergetic effect of Pb and iprodione (IPR) on adult zebrafish. Combined exposure to Pb and IPR disturbs zebrafish intestinal microbiota composition and disrupts metabolite profiles, weakening the gut barrier and provoking inflammation. This cascades via the gut–liver axis to promote liver injury through apoptotic pathways, with parental exposures further triggering transgenerational toxicity [95].

The microbiota that was altered in the zebrafish larvae group exposed for 60 days to a mixture of Pb and manganese (Mn) was associated with the gut–brain axis. Examination of the predicted functions of the microbiota revealed that pathways related to metabolites such as valine, leucine, isoleucine, D-alanine, and ketone bodies were markedly altered following combined exposure [97]. These metabolites are involved in neurological function and disorders including autism spectrum disorder and Alzheimer’s disease [111,112]. Lipid metabolism pathways (ABC transporters and fatty acid biosynthesis) were dysregulated in opposite directions between combined- and single-metal exposures, implicating altered host lipid homeostasis. Since lipid metabolic dysregulation is linked to neuropsychiatric disorders like schizophrenia [113], these findings highlight the metabolic contribution to neurotoxicity.

#### 2.4.2. Cadmium (Cd)

Cadmium (Cd) is a pervasive environmental pollutant classified as a human carcinogen with known bioaccumulative and neurotoxic properties.

Xia et al. studied microbiota changes in zebrafish larvae after short-term Cd exposure. Cd exposure caused a dose-dependent decrease in Firmicutes and an increase in Proteobacteria, a pattern resembling inflammatory bowel disease both in zebrafish models and humans [99]. At finer taxonomic resolution, reductions in beneficial taxa such as *Clostridia* and increases in *Gammaproteobacteria*, including opportunistic pathogens like *Pseudomonas*, were observed. Furthermore, some links to neurodegenerative diseases were identified. Decreased abundances of *Ruminococcaceae*, *Blautia*, *Bacteroides*, and *Lactobacillus* in Cd-exposed fish parallelled patterns linked to aging and neurodegenerative conditions like Parkinson’s [114] and Alzheimer’s diseases [115] in mammals. Increased *Pseudomonas* abundance correlates with neurological disorders such as multiple sclerosis and Creutzfeldt–Jakob disease [116]. Studies in nematode models show *Pseudomonas aeruginosa* infection induces hallmark neural degeneration [99]. To explore whether Cd-induced neurotoxicity is related to microbiota changes, Xu et al. established GF zebrafish larvae to Cd exposure. At a concentration of 0.01 mg/L (according to ecological standards in China), Cd inhibited the expression of several neuronal V-ATPase family genes, which are essential for lysosomal function. Notably, these toxic effects were significantly mitigated in GF zebrafish, highlighting the critical role of gut microbiota in mediating Cd neurotoxicity [32].

Zhang et al. investigated the combined effect of MP and Cd. It was found that MPs could transport Cd^2+^ into adult zebrafish via ciliate of the microbial loop. *Shewanella* was absent only in the MPs-Cd group, suggesting this species is sensitive to combined pollutant exposure. *Pseudomonas aeruginosa* and *Aeromonas veronii* populations increased with combined MP and Cd exposure. Xanthobacter sp., known for regulating immune homeostasis, decreased in both Cd and MP-Cd treatments, consistent with microbial community stress or injury [98]. Short-chain fatty acids (SCFAs) are known as critical mediators in neurodevelopment and neurotoxicity through the gut–brain axis. Exposure of larvae to ecologically relevant concentrations of Cd for 7 days revealed significantly disrupted gut microbial communities and alterations in SCFAs. Cd exposure reduced the relative abundances of eight gut genera linked to neurodevelopment. Acetic acid levels were positively associated with *Phascolarctobacterium* and *Candidatus saccharimonas*, genera known for producing neuroprotective SCFAs and which are implicated in improved cognitive function and psychiatric health [100].

Di et al. found that the effects of Cd were different in combined exposure with tetraconazole enantiomers and racemate (a mixture containing equal amounts (50:50) of the two enantiomers). Cd and tetraconazole, alone or combined, altered the gut microbiota of adult zebrafish, notably affecting key genera. *Cetobacterium* increased in the order Cd > Rac-tetraconazole > R-(−)- > S-(+)-tetraconazole, with Cd enhancing its abundance under S-(+)-tetraconazole exposure. *Edwardsiella* was promoted by S-(+)-tetraconazole but, with Cd, was inhibited in S-(+)- and promoted in R-(−)-tetraconazole treatments [101].

#### 2.4.3. Arsenic (As)

Exposure to arsenic—even at concentrations as low as 10 ppb, below the World Health Organization’s safety guidelines—significantly altered microbial diversity and community composition in larval zebrafish. Notably, 12 out of the 15 most abundant bacterial families constituting the healthy larval core microbiome exhibited significant abundance shifts upon arsenic exposure. Moreover, an increase the abundance of *int1* genes was observed in microbial communities; *int1* is an important genetic factor responsible for the transmission of gene cassettes conferring resistance to heavy metals and different antibiotics [103,117].

In the co-exposure of As (III) and fluoride at their environmentally relevant concentrations to the microbiota of zebrafish larvae, Proteobacteria dominated, with increases in beneficial phyla like Bacteroidetes and Firmicutes in combined exposure, potentially reflecting adaptive responses. Also, it was found that the presence of probiotic and immune-supporting genera (e.g., *Lactococcus*, *Phyllobacterium*) in the combined treatment group may have contributed to reduced toxicity, supporting the observed antagonistic effect. At the genus level, toxicant-specific enrichments were observed: fluoride treatment notably increased *Aeromonas*, which was linked to amplifying oxidative stress and tissue damage. Isolated *Aeromonas* appeared resistant to both As and fluoride and potentially contributory to fluoride-induced oxidative damage and immune modulation [105].

The study by Li et al. explored the co-exposure of adult zebrafish to As (V) and polystyrene nanoparticles (PSNPs). Exposure to As alone caused a decrease in overall bacterial diversity, whereas combined As+PSNP treatment surprisingly led to increased gut biodiversity. Both As and As+PSNP treatments caused an increase in Proteobacteria, a phylum enriched in lipopolysaccharide-producing bacteria which can damage the intestinal barrier; an increase in permeability; and the triggering of inflammation [118]. Firmicutes, beneficial for energy absorption, anti-inflammatory effects, and lipid metabolism, decreased with pollutant exposure, indicating potential compromised host metabolic and immune functions. The abundance of *Actinobacteriota*, important for synthesizing antimicrobial secondary metabolites and enhancing fish immunity, was markedly reduced in combined exposures, suggesting increased vulnerability to oxidative stress and gut barrier disruption. At the genus level, beneficial *Cetobacterium* was increased, possibly reflecting its role in helping withstand environmental stress. Conversely, *Lactobacillus* and *Bifidobacterium* were significantly reduced in the co-exposure group, implying impaired immune defenses and heightened oxidative stress. The genus *Rhodococcus*, known for pathogenicity in fish, increased under co-exposure, suggesting PSNPs may exacerbate inflammatory conditions [104].

#### 2.4.4. Chromium (Cr)

Zebrafish embryos were exposed to 1 mg/L Cr (III) or Cr (VI) for 7 days. The phylum-level changes included decreased Bacteroidetes abundance and variable effects on Firmicutes: increased with Cr (III) and decreased with Cr (VI) exposure. At the genus level, an increase in *Clostridium sensu stricto 13* (Firmicutes) in Cr (III)-exposed fish was notable. Increased *Clostridia*-derived secondary bile acids can induce host serotonin production, linking bile acid metabolism to neurochemical signaling. Dysregulation of bile acids and gut microbiota compositions have been documented in human and mouse model neurodegenerative diseases like Parkinson’s, Alzheimer’s [119], and multiple sclerosis [120]. An increase in *Cetobacterium* was observed in Cr (III)-exposed fish [102].

#### 2.4.5. Metal Nanoparticles

**Nanoparticles** (NPs) are being increasingly utilized across a range of products and industries, notably in water treatment and disinfection applications. In biomedical contexts, NPs are classified into several types: metallic NPs like gold and silver; bimetallic or alloy NPs such as iron–cobalt and iron–platinum; metal oxide NPs including titanium dioxide, cerium dioxide, silica, and zinc oxide; and magnetic NPs, with iron oxide being a prominent example [121]. In aquatic ecosystems, NPs are primarily derived from industrial processes, such as manufacturing and wastewater treatment, as well as from consumer products like cosmetics and sunscreens [122]. Recent studies have indicated that ENP concentrations in aquatic environments vary from nanograms to micrograms per liter [123]. Some NPs such as silver, zinc oxide, titanium dioxide, copper, and iron oxide particles have antimicrobial properties; these metal NPs have the ability to disrupt cellular membranes, DNA, and proteins through direct physical contact or by triggering the generation of reactive oxygen species [124]. However, NPs can interfere with microbiota and lead to dysbiosis.

Short-term exposure to silver nanoparticles (nAg) and silver ions (Ag^+^) resulted in a dramatic reduction in culturable bacteria associated with zebrafish larvae, leaving nearly no viable microbes after two days. In contrast, neither zinc oxide nanoparticles (nZnO) nor zinc ions (Zn^2+^) at sublethal levels affected the bacterial abundance in zebrafish larvae. Further analysis of microbial communities showed that, prior to nAg exposure, opportunistic bacteria such as *Pseudomonas aeruginosa*, *Deinococcus lacustris*, *Deinococcus tsuruhatensis*, *Stenotrophomonas maltophilia*, and possibly *Staphylococcus epidermidis* were present. After treatment with nAg, these species were absent. Remarkably, *Pseudomonas myrsinacearum* was the only microbial isolate showing resistance to nAg, a trait that might be linked to its ability to convert Ag^+^ ions into less toxic forms via nitrate reduction [125,126]. Continuous nAg exposure reduced microbial diversity and richness while driving the gut microbiota in adult zebrafish toward an alternative, less stable state marked by proliferation of opportunistic pathogens like *Citrobacter* and increased Proteobacteria, an indicator of dysbiosis. Key microbial taxa such as *Acinetobacter* and *Gemmata* were vital for maintaining network integrity and supporting resilience by aiding digestion and resisting harsh conditions. Although the gut microbiota partially rebounded after removal of AgNP exposure, complete restoration to the original community structure was not achieved [107].

Acute exposure to copper oxide nanoparticles (CuO NPs) in zebrafish larvae leads to an increased relative abundance of Proteobacteria and a decrease in both Firmicutes and Bacteroidetes when compared to controls, reflecting a notable disturbance in the core microbiome, which is a hallmark of inflammatory bowel conditions in both zebrafish and humans. Further microbiome analyses reveal that specific taxa such as *Arsenicibacter* and *Flavobacteriales* increase significantly at lower nanoparticle concentrations, while beneficial bacterial groups, including butyrate-producing *Lachnospiraceae* and *Syntrophomonadaceae*, decrease across treated groups. Furthermore, changes are observed in taxa associated with immune modulation—such as an increase in *Corynebacteriaceae* and a decrease in *Shewanella*, the latter known for dampening inflammation [127]. CuO NP exposure also changed the abundance of gut microbes involved in metabolizing SCFAs and lipopolysaccharides, decreasing levels of acetic, propionic, and valeric acids [128].

Another study investigated how engineered nanoparticles affect gut microbiota throughout the zebrafish life cycle (from larvae to adulthood). Nanoparticle exposure reduced zebrafish gut microbial diversity after 53 days post-hatching and altered community composition and structure. Gut microbiota assembly was primarily driven by homogeneous selection linked to development and exposure. Nanoparticles also decreased network modularity and increased positive interactions, indicating a less stable microbial community [106].

#### 2.4.6. Dyes

Chronic exposure to textile wastewater treatment plant (TWTP) effluent caused developmental toxicity and growth inhibition in adult zebrafish, as indicated by decreased condition factor (K-factor), higher mortality rates, and histopathological damage to gut and liver tissues. Gut and water microbial diversity increased in response to TWTP effluent, potentially raising the risk of opportunistic pathogen colonization. This was evidenced by elevated abundances of pathogenic genera such as *Escherichia*, *Vibrio*, and *Legionellales*, suggesting a greater chance of intestinal inflammation in exposed fish. Increased *Cetobacterium* was also observed and may have related to ammonia stress. At the phylum level, Proteobacteria increased, indicating disrupted energy metabolism and gut microbial instability, while Bacteroidetes expanded, possibly influencing host energy balance and body weight fluctuations. Functional prediction revealed upregulation of genes involved in amino acid biosynthesis, arginine/proline metabolism, and the pentose phosphate pathway, alongside downregulation of carbohydrate metabolism pathways, suggesting disruption of zebrafish digestion and metabolism. These molecular changes may contribute to impaired growth and development observed during chronic exposure [129].

#### 2.4.7. Microplastics

Microplastics (MPs) have emerged as a significant environmental contaminant, with global plastic waste generation estimated at around 6.30 billion tons from 1950 to 2015, of which approximately 80% accumulates in the natural environment [130]. As Mani et al. reported, microplastics are found throughout major waterways, with their study of the Rhine River revealing an average of 892,777 microplastic particles per km^2^, and concentrations reaching as high as 3.9 million particles per km^2^ in metropolitan areas. These particles originate from various sources including fragmentation of larger plastic debris, pre-production pellets, and components of consumer and industrial products, with an estimated 80% of marine plastic pollution originating from inland sources transported by rivers [131]. Microplastic contamination was ubiquitous across all effluent samples, exhibiting size-dependent concentration patterns: larger particles (>500 μm) ranged from 0 to 5 × 10^1^ m^−3^, while smaller particles (<500 μm) showed higher concentrations of 1 × 10^1^ to 9 × 10^3^ m^−3^. Polyethylene emerged as the dominant polymer across both size fractions, whereas synthetic fibers, present at concentrations of 9 × 10^1^ to 1 × 10^3^ m^−3^, were predominantly composed of polyester [132].

MPs, which can range from 0.1 to 5000 μm, are especially problematic due to their high surface area and hydrophobic character, allowing them to absorb various environmental pollutants including heavy metals [133], polycyclic aromatic hydrocarbons, and polychlorinated biphenyls [134]. Once absorbed, these contaminants can be transferred to aquatic organisms, subsequently entering the food chain [135].

All revised studies related to MP exposure are summarized in Table 4.

Studies have shown that microplastics can adhere to fish skin or translocate to various tissues including gills, liver, and muscle, with very fine particles capable of penetrating multiple organ systems [146]. Recent studies have also demonstrated that MP exposure can significantly alter the gut microbiome of aquatic species. For instance, research has shown that MP exposure can change the gut microbiome of silver carp and alter host metabolism, particularly amino acid metabolism, along the gut–liver–muscle axis [147]. This disruption to the gut microbiome may lead to altered intestinal structure and function, including changes in permeability, inflammatory and immune responses, and oxidative stress. In zebrafish, microplastics have been detected in numerous tissues and organs including the intestinal tract and other organs [148]. The intestinal tract represents a critical site of microplastic accumulation in zebrafish, where these particles can directly interact with the gut microbiota and intestinal epithelium.

Multiple studies have documented significant alterations in the gut microbiome of zebrafish following microplastic exposure. Jin et al. reported that polystyrene microplastics (0.5 and 50 μm) at concentrations of 100 and 1000 μg/L significantly disrupted microbial communities in adult zebrafish after 14 days of exposure. At the phylum level, they observed decreased abundances of Bacteroidetes and Proteobacteria with concurrent increases in Firmicutes. High-throughput sequencing of the 16S rRNA gene revealed significant changes in both richness and diversity of the gut microbiota, with 29 operational taxonomic units significantly altered in both treatment groups [136]. In addition to that, Qiao et al. demonstrated that different kinds of microplastics in adult zebrafish guts also led to significant changes in their intestinal tracts. These included multiple toxic effects in the intestine, that included mucosal damage, increased permeability, inflammation metabolism disruption, and bacterial dysbiosis [137].

Kurchaba et al. showed that prolonged polyethylene MP exposure in the early life of zebrafish did not lead to any severe consequences in the metabolism or immune system of the fish; however, it led to local gut damage by oxidative stress and disturbance of the gut microbiome [138]. Conversely to Jin et al., they documented significant disruptions to the microbiome’s composition, with an increased abundance of *Bacteroidetes*—a pattern frequently associated with intestinal pathologies. Despite the dominance of Proteobacteria (90%) across all samples, they identified a core microbiome consisting of 17 genera, including *Aeromonas*, *Pseudomonas*, and *Vibrio*, which showed varying responses to microplastic exposure. Medriano and Ba demonstrated increased relative abundances of Fusobacteria and Proteobacteria with concurrent declines in Firmicutes after exposing adult zebrafish to environmentally relevant concentrations of polyethylene (PE) and polyester (PES). Microbial diversity analyses indicated microbiota dysbiosis alongside metabolomic dysregulation and oxidative stress, suggesting that even acute exposure to environmentally relevant concentrations of microplastics could disrupt metabolic interactions via the gut–liver–brain axis [139]. Li et al. found that chronic exposure to PE microplastics and nanoplastics significantly altered the gut microbiota composition of adult zebrafish, with the average relative abundance of Proteobacteria increasing from 29.73% (control group) to 66.10% (microplastics), 54.84% (nanoplastics), and 60.03% (combined exposure), while Tenericutes decreased from 55.43% (control group) to 20.02% (microplastics), 22.44% (nanoplastics), and 31.77% (combined exposure) [140].

Choi et al. examined the effects of both virgin polypropylene microplastics (PP) and UV-weathered PP (UV-PP) at 50 mg/L for 14 days on the microbiome of adult zebrafish, finding alterations in the abundance and diversity of gut microorganisms, with more pronounced changes observed in the virgin PP-exposed group. Their pathway analysis demonstrated associations between these microbial alterations and cellular responses including oxidative stress, inflammation, and tissue damage [141].

The size of microplastic particles appears to influence the severity of gut microbiome disruption. Jin et al. observed that smaller microplastics (0.5 μm) induced stronger inflammatory responses compared to larger particles (50 μm), suggesting size-dependent effects on gut health [136]. Similarly, Choi et al. found that UV weathering made polypropylene microplastics smaller and increased their accumulation in the zebrafish gut, potentially affecting microbial communities through different mechanisms than virgin microplastics [141].

Mancia et al. investigated the effect of pristine (PEv) and previously incubated PE (PEi) microplastics administrated with food to adult zebrafish. Significant shifts occurred at all phylogenetic levels, demonstrating that both PEv and PEi can uniquely alter the dominant bacterial taxa in the intestine. In the PEv group, the microbiome was marked by a higher abundance of *C. somerae* and *Bacillus spongiae*—reflecting dominance of the *Fusobacteriaceae* and *Bacillaceae* families. *C. somerae* is known to be a beneficial symbiont in marine animals, fermenting proteins and peptides into short-chain fatty acids useful for both bacteria and host [143]. The findings of increased *C. somerae* with PEv exposure align with prior studies showing similar trends in aquatic species exposed to plastic or related pollutants [149]. The gut microbiome of fish exposed to PEi showed a distinct composition compared to the PEv group, featuring elevated levels of *Bacillus tianshenii*, *Desulfofrigus fragile*, and *Haloplasma contractile*. These taxa could be involved in colonizing or degrading plastics and related compounds. Additionally, all signs of mucosal distress worsened in fish exposed to PEi- than to PEv-MPs. The introduction of incubated MPs, which carry environmental biofilm and associated micropollutants, may foster the integration of external plastisphere bacteria into the gut community—overriding typical endogenous microbiome responses observed with virgin MPs [143].

Beyond gut effects, studies have observed broader physiological impacts. Li et al. found inhibited acetylcholinesterase (AChE) activity, suggesting potential neurotoxicity from both microplastics and nanoplastics [140]. Qiang et al. investigated the transgenerational effects of microplastics in adult zebrafish and found that continuous exposure to high concentrations (>100 μg/L) of polystyrene microplastics (1 μm) for 21 days resulted in notable microplastic accumulation in adult fish intestines and induced significant changes in steroidogenic mRNA expression in zebrafish gonads. However, no significant changes were observed in egg production, fertilization rate, or early development of offspring, suggesting transgenerational effects might be negligible or recoverable [142].

Duan et al. compared the effect of petroleum-based (polyethylene terephthalate (PET)) and bio-based (polylactic acid (PLA)) microplastics. Exposure of adult zebrafish to PET microplastics caused notable alterations in the zebrafish intestinal microbiota, consistent with previous findings. Interestingly, while considered to be “eco-friendly”, PLA MPs caused greater disturbances to the gut microbiome compared to PET MPs, correlating with intensified intestinal damage and inflammation. At the phylum level, shifts in Proteobacteria, Firmicutes, and Bacteroidetes were more pronounced, alongside the emergence of Tenericutes and Acidobacteria—phylotypes associated with severe fish diseases and inflammation. At the genus level, *Mycoplasma* replaced *Cetobacterium* as the dominant taxon in PLA-treated fish. Both PET and PLA microplastics disrupted the zebrafish gut microbiota, but PLA MPs had a more profound impact—shifting dominant microbial populations toward inflammation-associated taxa and inducing stronger dysbiosis—potentially mediated by PLA degradation products such as lactic acid [144].

Medriano et al. investigated the chronic exposure of PES and PE microplastics in adult zebrafish for 30 days. It was found that PES fibers caused more severe intestinal toxicity than PE fragments, mainly due to longer intestinal residence time and the nature of fiber, disrupting gut microbiota more significantly. Chronic PE and PES exposure induced shifts in the gut microbial communities of zebrafish, with potential increases in pathogenic bacteria and alterations in commensal bacteria: increased prevalence of potential pathogens like *Mycobacterium* (notably in PES exposure) and *Aeromonas* (in both PE and PES) was observed, raising infection risk. Co-occurrence network analysis showed increased complexity of microbial interactions after chronic MP exposure, suggesting adaptive responses maintaining microbial ecosystem stability despite dysbiosis [145].

Micro- and nanoplastic exposures consistently disturb the zebrafish intestinal ecosystem. Across polymer types (PS, PE, PP, PES) and particle sizes (≤0.5 µm to 50 µm), plastics accumulate along the mucosa, erode epithelial barriers, and drive characteristic dysbiosis. Multi-omics links these shifts to suppressed amino acid and lipid pathways, oxidative stress, and gut–liver–brain inflammatory signaling, while finer or UV-weathered fragments provoke the strongest responses. Together, these data frame the zebrafish gut microbiome as both an early bio-indicator and a mechanistic hub for systemic microplastic toxicity.

Key blind spots remain. Most experiments still rely on mg/L doses that dwarf environmental levels, and systematic comparisons of nanoplastics, aged particles, or mixed polymers are scarce. Long-term and multigenerational consequences are virtually unexplored, as is the capacity of plastics to vector co-contaminants and compound microbiome injury. Functional work rarely couples deep sequencing with metabolomics or host immuno-phenotyping, hindering causal inference. Progress now depends on environmentally realistic exposures, chemically verified particles, harmonized bioinformatic pipelines, and multi-omics designs that can unravel whether microbiome disruption is merely a sentinel—or a driver—of organism-level toxicity.

## 3. Taxa Associated with Gut Dysbiosis in Zebrafish

**Figure 1:**
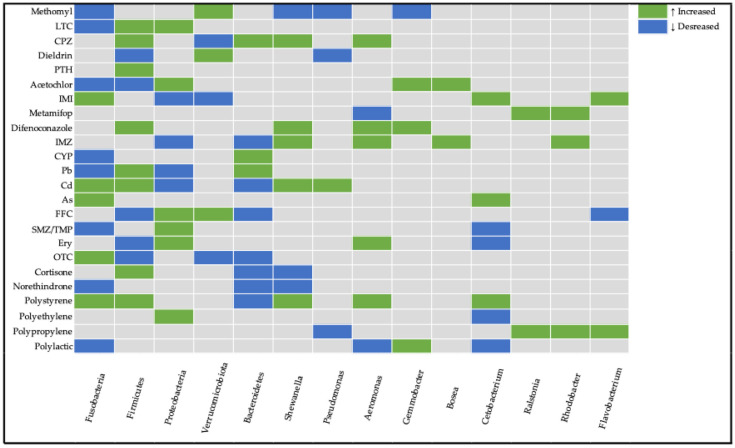
Heatmap visualization of bacterial community responses to environmental pollutants across the reviewed literature. Color coding represents directional abundance changes: green for upregulation, blue for downregulation, and gray for unchanged populations compared to control conditions.

The taxonomic relationships observed across the included studies are detailed in Table 5 and visualized in Figure 1, which synthesize contaminant-induced alterations in bacterial phyla and genera within the zebrafish gut microbiome. In Table 5 and Figure 1, only studies conducted on adult zebrafish are included. Only microbial features at the phylum and genus level were considered and reported for this review (Table 5 and Figure 1). At the phylum level, the gut microbiome of zebrafish comprises four dominant phyla, including Proteobacteria, Fusobacteria, Firmicutes, and Bacteroidetes, followed by some less present, such as Gemmatimonadetes, Cyanobacteria, and Verrucomicrobiota. Among the studies included in this review, the bacterial phyla most frequently reported to undergo contaminant-induced changes were Proteobacteria, Firmicutes, Fusobacteria, Bacteroidetes, and Verrucomicrobiota.

Proteobacteria show the most widespread responses, with both increases and decreases across multiple contaminants. Firmicutes demonstrate variable responses, tending to be positively affected depending on the contaminant type. Antibiotic exposure tends to cause a decrease in the abundance of Firmicutes and an increase in the abundance of Proteobacteria. Fusobacteria and Verrucomicrobiota show more selective responses to specific contaminants.

Some notable genera which we encountered often among the reviewed studies are *Shewanella*, *Pseudomonas*, *Aeromonas*, *Gemmobacter*, *Bosea*, *Cetobacterium*, *Ralstonia*, *Rhodobacter*, and *Flavobacterium*. At the genus level, changes in *Aeromonas*, *Pseudomonas*, and *Shewanella* were observed across multiple contaminants, often reflecting opportunistic growth under stress conditions. *Rhodobacter*, considered a beneficial bacteria, was increased in three studies. *Bosea* and *Ralstonia* increased in pesticide-induced dysbiosis. Other genera such as *Cetobacterium* had both directions of modulation in different studies.

These findings demonstrate that contaminant exposure influences gut microbial composition in both taxa-specific and contaminant-specific manners. While some general trends can be identified, each pollutant appears to exert distinct effects on the zebrafish gut microbiome.

## 4. Discussion

### 4.1. Role and Functions of Altered Phyla and Genera in Gut Microbiome of Zebrafish

Zebrafish provide a unique and convenient in vivo model for investigating vertebrates’ microbiome alterations in toxicological studies of environmental pollutants. Zebrafish possess intricate gut microbial communities that are susceptible to disruption by diverse environmental contaminants including antibiotics, microplastics, nanoplastics, pesticides, and dyes. At the phylum level, the gut microbiomes of zebrafish comprise four dominant phyla, including Proteobacteria, Fusobacteria, Firmicutes, and Bacteroidetes, followed by some that are less present, such as Gemmatimonadetes, Cyanobacteria, and Verrucomicrobiota. The abundance of these features often shifts across studies, with multiple bacteria showing conflicting findings between said studies (Table 5 and Figure 1). While the levels of each bacterium within the gut are not conclusive in nature, their sudden shifts in abundances between control and treatment groups are vital.

The phylum **Proteobacteria**, including *Aeromonas*, *Pseudomonas*, and *Vibrio* genera, is the most abundant in zebrafish. Members of the Proteobacteria phylum are predominantly associated with nitrogen metabolism and carbon compound binding processes. Changes in their abundance might serve as biomarkers for intestinal microbiome disruption in fish hosts. Decreased abundance can compromise the host’s metabolic regulatory processes and digestive efficiency [150]. Conversely, elevated levels may trigger lipopolysaccharide synthesis, resulting in inflammatory responses, intestinal barrier dysfunction, and enhanced gut permeability [118,151]. Additionally, elevated abundances of Proteobacteria are regularly observed in individuals with comprised health [152]. In the 4 of 9 studies where alterations in Protobacteria were reported, their abundance was elevated, although this trend was not found in all studies (Table 5 and Figure 1.).

**Firmicutes** are predominantly acknowledged for their growth-promoting properties; these microorganisms assist in optimizing nutrient assimilation, as well as influencing the metabolism of glucose, lipids, and energy of the host [2,153,154]. They are also major participators in fatty acid absorption [155]. Decreased levels may impair nutrient uptake and have been identified as a characteristic biomarker of IBD-like colitis [127] in specific fish species, though elevated bacterial populations have also been associated with obesity development [156]. In Figure 1 the trend of increasing Firmicutes is noticeable.

**Verrucomicrobiota** are mucin-degrading bacteria; their presence is considered to regulate gut health [157]. Research also links their decline to reduced growth rates while associating their increase with enhanced body weight [158]. In some reviewed studies, Verrucomicrobiota were increased (Figure 1). They might serve as an adaptation to stress or inflammation.

The phylum **Bacteroidetes** is another common member of the intestinal microbiome. It serves a vital role in preserving the dynamic equilibrium of the intestinal mucosal immune system [159]. It also plays a significant role in dietary digestion through its participation in protein metabolism and carbohydrate transport processes [160]. The phylum also shows associations with gene expression related to nutrient absorption, metabolism, and strengthening the mucosal barrier [156]. Trends suggest that elevated Bacteroidetes levels provide advantageous effects to the host, functioning not as a marker of wellness, but as a protective response to mitigate challenges facing the host.

Some notable genera which we encountered often among the reviewed studies are *Shewanella*, *Pseudomonas*, *Aeromonas*, *Gemmobacter*, *Bosea*, *Cetobacterium*, *Ralstonia*, *Rhodobacter*, and *Flavobacterium*.

*Shewanella* is an opportunistic pathogen capable of infecting diverse aquatic organisms [161]. Nevertheless, research has also emphasized its beneficial properties, particularly its association with plastic biodegradation of materials, including low-density polyethylene and polycaprolactone [162]. In the majority of reviewed studies, an increase in *Shewanella* abundance may have served as a sign of pathogenic microbiota proliferation. *Cetobacterium* might be considered probiotics; they possess the capability to synthesize vitamin B12 [163], which enhances overall fish nutrition; stimulate insulin expression that reduces blood glucose levels in fish [164]; and generate antimicrobial metabolites that can strengthen the fish immune system [139]. In the majority of studies, *Cetobacterium* (Table 5 and Figure 1) were reduced, which aligns with comprised health.

*Bosea* represents a potential key biomarker for the antidepressant activity of adipose-derived mesenchymal stem cells in mouse models subjected to chronic social defeat stress [109]. *Bosea* species have also been demonstrated to express an enzyme capable of degrading N-acyl homoserine lactones (AHLs) utilized in quorum sensing mechanisms. AHLs are employed by pathogenic bacteria to regulate density-dependent virulence factors, and the disruption of these quorum-sensing molecules in pathogens could consequently provide beneficial effects for host health [165].

*Rhodobacter* might be considered beneficial bacteria. Some members of this genus were found to reduce Pb accumulation and be linked to secondary bile acid synthesis [166]. It has been reported to be increased in three studies, which may be linked to the adaption of the gut microbiome to pollutant-induced stress.

*Pseudomonas*, *Flavobacterium*, *Aeromonas*, and *Ralstonia* are commonly found within fish with pathogenic functions which pose health risks [167]. In the majority of studies where these genera were found to be increased, it might have been associated with attenuated immune function (Figure 1).

*Gemmobacter*, a characteristic anaerobic denitrifying bacterium, is linked to organic matter metabolism and demonstrates capacity for degrading environmental pollutants [168]. Intriguingly, this genus was enhanced in some studies, and might be a sign of response to pollutant exposure.

### 4.2. Gut Microbiome Response of Zebrafish to Contaminant Exposure

Pesticides have varying impacts on the organism, gut microbiota, and metabolic pathways in zebrafish. They alter the composition and diversity of the gut microbiota, resulting in imbalances among bacterial groups, metabolic disturbances, impaired lipid absorption, inflammation, oxidative stress, changes in liver metabolism, and disruptions to gut barrier function. Hence, exposure to compounds such as methomyl, cyproconazole, acetochlor, imidacloprid, and penthiopyrad can lead to upregulation of inflammatory gene expression together with intestinal injury, such as villi shortening and breakage or their atrophy. Oxidative stress was observed in intestinal tissue after exposure to λ-cyhalothrin, metamifop, and imidacloprid. Additionally, chronic exposure of methomyl resulted in a stress response—cortisol overexpression and apoptosis activation. Specific metabolic disturbances in lipid metabolism were indicated for compounds such as λ-cyhalothrin—lipid peroxidation, metamifop—impaired fat absorption, difenoconazole—disorders of lipid metabolism, and liver function. Intriguingly, the pesticide cypermethrin, apart from gut dysbiosis, also enhanced ARG complexity. Pesticides cause shifts in the composition of the microbiota, often accompanied by the loss of “beneficial” and the growth of “harmful” microorganisms: for example, the reduction in Firmicutes (associated with energy supply), Bacteroides, Verrucomicrobia (important for the synthesis of fatty acids and maintaining immunity), *Psychrobacter*, and *Aeromonas* (support for immunity and digestion). Meanwhile, the increase in *Proteobacteria* is often correlated with inflammation, intestinal integrity disorders, oxidative stress, and metabolic disruptions.

Antibiotics can cause dysbiosis in zebrafish by disturbing gut balance, changing the composition of the microbiota, and promoting the growth of pathogenic bacteria as well as antibiotic resistance genes. Different types of antibiotics have differential targets, affecting microbial composition, and can affect metabolism and immunity. ENR belongs to the fluoroquinolone antibiotic; the mechanism of action is the inhibition of nucleic acid synthesis. ENR causes a disruption of the microbiota together with immunosuppression (a decrease in macrophages and neutrophils). Antibiotic exposure leads to activation of ARG and enhances the risk of intestinal inflammation. The consumption of antibiotics reduces susceptibility to some bacterial infections (*Edwardsiella piscicida*), but at the same time increases susceptibility to other pathogens (*Aeromonas hydrophila* and viruses). Antibiotics not only reduce pathogens, but also stimulate the growth of certain “beneficial” or opportunistic groups (for example, *Rhizobiales*). Additionally, shifts in the microbiota are accompanied by a decrease in beneficial bacteria (for example, *Pediococcus*) and an increase in pathogenic or inflammatory-associated genera (*Ralstonia*, *Escherichia shigella*, *Aeromonas*).

Exposure to heavy metals (lead, cadmium, arsenic, chromium) and metal nanoparticles disrupts the gut microbiota of zebrafish, causing dysbiosis characterized by increased Proteobacteria and decreased beneficial taxa such as Firmicutes, *Actinobacteria*, and Bacteroidetes. These microbial shifts are linked to altered metabolic pathways, including amino acid and short-chain fatty acid metabolism, which are crucial for neurodevelopment and immune function. Dysbiosis contributes to intestinal barrier dysfunction, inflammation, and neurobehavioral impairments. Metal nanoparticles further reduce microbial diversity, promote opportunistic pathogens, and impair gut microbial resilience.

MPs are harmful alone but also can adsorb pollutants like heavy metals and organic toxins, facilitating their transfer to aquatic organisms and entry into the food chain. In zebrafish and other fish species, MPs accumulate in various tissues, notably the gut, where they disrupt the intestinal microbiome. Exposure to MPs causes significant alterations in microbial community composition, including shifts in key bacterial phyla such as decreased Bacteroidetes and Proteobacteria and increased Firmicutes and Fusobacteria, accompanied by reduced diversity and dysbiosis. These changes lead to impaired gut barrier function, inflammation, oxidative stress, and disturbances in host metabolism along the gut–liver–brain axis. The severity of the effects depends on microplastic size, type, weathering status, and exposure duration, with smaller and UV-weathered particles generally inducing stronger impacts. Chronic exposure to different plastic polymers (e.g., polyethylene, polypropylene, polyester) further modifies microbial communities, increasing pathogenic bacteria and intestinal toxicity. Although some adaptive microbial responses help maintain community stability, the overall impact contributes to physiological dysfunction, including potential neurotoxicity and reproductive effects.

Overall, despite advances, current research often uses unrealistically high exposure levels of contaminants, with limited investigation into long-term, multigenerational consequences, and the role of plastics as vectors for co-contaminants. Future studies employing environmentally relevant conditions and multi-omics approaches are essential to clarify whether microbiome disruptions are biomarkers or causal factors of microplastic toxicity. Future studies should prioritize investigating the sustained biological impacts of freshwater pollutants, emphasizing chronic health outcomes across multiple physiological domains.

### 4.3. Differences in Microbiome Alterations in Adult and Larvae Zebrafish

Zebrafish gut microbial communities undergo predictable, development-dependent changes: as hosts mature from larval to adult stages, microbial diversity, interspecies interactions, and ecological network stability all increase [169]. Developmental stage is a critical factor shaping how the zebrafish gut microbiome responds to environmental pollutants. In early life stages (embryos and larvae), the gut microbiome is still establishing and tends to have lower diversity and functional redundancy, making it more susceptible to perturbation by toxicants. Pollutant exposure during this period can cause long-lasting shifts in community composition and functional capacity, potentially due to disruption of microbiome assembly and host immune system development.

Lead exposure induces markedly different microbiome responses across zebrafish developmental stages. Larval stages (5–7 days post-fertilization (dpf)) demonstrate heightened microbiome sensitivity, with profound neurodevelopmental implications, characterized by significant bacterial composition shifts and potential long-term neurological consequences. In contrast, adult stages exhibit more nuanced microbiome alterations, primarily manifesting as metabolic and hepatic disruptions [94,96].

Arsenic exposure induces stage-specific microbiome alterations in zebrafish, with even low concentrations (10 ppb) significantly restructuring microbial communities. Larvae demonstrate increased genetic plasticity through elevated *int1* gene abundance and proliferation of arsenic-resistant taxa, while adult zebrafish show metabolic responses characterized by shifts in bacterial populations and increased oxidative stress. These developmental-stage-dependent changes reveal complex microbiome interactions, suggesting that arsenic impacts are nuanced and context-dependent. The findings highlight the critical importance of considering developmental stage when assessing environmental contaminant effects on host–microbiome dynamics [98,99].

Early-life exposure to low antibiotic levels may promote pathogenic *Flavobacterium* accumulation, potentially predisposing zebrafish to subsequent health complications as has been noticed in the co-exposure of zebrafish to SMZ and OTC from the larvae stage to adulthood [68].

Greater microbial disruption at 9 dpf than 15 dpf was observed with PE-MP, suggesting life stage-sensitive dysbiosis. Increased Bacteroidetes were present at both life stages, and decreased *Actinobacteria* present at 15 dpf. Compared to adult zebrafish exposed to PE-MPs, changes in the microbiome were slightly increased in Proteobacteria and reduced in Tenericutes, while the dominant phyla set (Proteobacteria, Firmicutes, *Actinobacteria*, Fusobacteria) remained the same [138].

Adults show adaptive microbiome responses with increased diversity and plastic-degrading bacteria, while larvae exhibit stress-induced dysbiosis with oxidative damage and reduced resilience during developmental transitions.

## 5. Conclusions

In summary, studies on zebrafish have demonstrated that exposure to a wide range of environmental pollutants—including pesticides, microplastics, heavy metals, antibiotics, dyes, and other contaminants—adversely affects gut health, disrupts gut microbiota composition, and impairs metabolic functions. Taking into account the evidence that the gut microbiota can affect various systems beyond the intestines, such as the liver or nervous system, it is crucial to study microbiome in ecotoxicological studies.

This review is subject to several limitations. First, for many pollutants, available evidence is limited to a single study, which restricts the strength of any conclusions and prevents a detailed comparative analysis across studies. Second, some studies used concentrations that exceed environmental concentrations. Third, exposure conditions and zebrafish developmental stages examined varied widely among studies, which complicates direct comparisons and may contribute to heterogeneous or conflicting results.

Studies mentioned in this review highlight the urgent need for comprehensive environmental monitoring and the development of effective mitigation strategies, alongside deeper investigation into the mechanisms driving these toxic effects.

## 6. Methods

### Data Collection

For the narrative review we systematically explored the scientific literature through PubMed^®^ and Scopus^®^ databases as well as “Google Scholar” searches. To identify relative studies, the following keywords were applied: “Danio rerio & microplastic & microbiome”, “Danio rerio & pesticide & microbiome”, “Danio rerio & heavy metal & microbiome”, “Danio rerio & hormones & microbiome”, “Danio rerio & antidepressants & microbiome”, “Danio rerio & dye & microbiome”, “Danio rerio & environmental pollutants & microbiome”, and “Danio rerio & xenobiotics & microbiome”. Scopus^®^ yielded 81 records, many of which were directly relevant to the review’s focus. PubMed^®^ returned 400 records, providing a broader range of articles but with lower overall relevance. Google Scholar^®^ produced more than 10,000 hits; given the large volume and algorithm-based ranking, we screened only the first 100 most relevant results to identify any studies not captured in the other databases. Inclusion criteria were as follows: original experimental studies using Danio rerio as the model organism; research assessing gut microbiome composition after exposure to at least one environmental contaminant; and publication in English in peer-reviewed journals. Exclusion criteria were as follows: reviews, conference abstracts, book chapters, or non-peer-reviewed sources; studies on pathogenic gut organisms without pollutant exposure; and work not involving Danio rerio or not reporting microbiome data. Eventually, after exclusion of irrelevant articles and removal of duplicates, we included 52 original articles in our analysis.

## Figures and Tables

**Table 1:** Experimental conditions for pesticide exposure studies using zebrafish models: pesticide types, concentrations, and exposure durations.

Contaminant	Zebrafish Model	Concentration	Days of Exposure	Ref.
Methomyl	juvenile	0.05, 0.10, and 0.20 mg/L	56	[41]
λ-cyhalothrin	adult	0.1 μg/L	21	[42]
Cyproconazole	adult	50 and 500 μg/L	40	[43]
Dieldrin	adult	16 ng/g	~120	[44]
Penthiopyrad	adult	0.03 mg/L	21	[45]
Acetochlor	adult	0.05 mg/L	21	[46]
Imidacloprid	adult	1 mg/L	14	[47]
Metamifop	adult	0.025, 0.10, and 0.40 mg/L	21	[48]
Imidacloprid	adult	1000 μg/L	21	[49]
Difenoconazole	adult	0.4 mg/L	21	[50]
Imazalil	adult	1000 μg/L	21	[51]
Cypermethrin Polystyrene	adult	2.5 μg/L and 500 μg/L	21	[52]

**Table 2:** Experimental conditions for antibiotics and other medicine exposure studies using zebrafish models: medicine types, concentrations, and exposure durations.

Contaminant	Zebrafish Model	Concentration	Days of Exposure	Ref.
Antibiotics				
Oxytetracycline; sulfamethoxazole/trimethoprim; erythromycin	adult	1 mg/L	30	[62]
Florfenicol	adult	15 mg/kg of body weight per day	10	[63]
	adult	5, 10, 20, 40 mg/L	56	[64]
Oxytetracycline	adult	100 μg/L	30	[65]
Enrofloxacin	larvae	0.01, 0.1, 1, 10, 100 μg/L	~60	[66]
Doxycycline; oxytetracycline; florfenicol	adult	10, 30, 100 μg/L	21	[67]
Sulfamethoxazole; oxytetracycline	from larvae to adult stage	1000 ng/L; 5000 ng/L	~120	[68]
Oxytetracycline	adult	10, 10,000 μg/L	~60	[69]
Streptomycin	adult	0.1, 1, 10 mg/L	10	[70]
Tetracycline	juvenile	1, 100 μg/L	~30	[71]
Sulfamethoxazole; clarithromycin	larvae	0.01 mg/L; 0.01 mg/L;	14	[72]
Other medicines				
Amitriptyline	adult	2.5, 10, 40 μg/L	7	[73]
Cortisone	adult	5, 50, 500 ng/L	7	[74]
Norethindrone	adult	~70 ng/L	30	[75]

**Table 3:** Experimental conditions for heavy metal exposure studies using zebrafish models: heavy metal types, concentrations, and exposure durations.

Contaminant	Zebrafish Model	Concentration	Days of Exposure	Ref.
Lead	larvae	0.05 mg/L	3	[94]
	adult		60	[95]
	adult	10 and 30 μg/L	7	[96]
Lead + manganese	larvae	Pb: 0.05 mg/L; Mn: 0.3 mg/L	7	[97]
Cadmium	larvae	25 µM, 100 µM	7	[32]
Cd and MPs	adult	Cd^2^⁺: <0.1 µg/L; MPs: 1 × 10^5^ items/L	2	[98]
	larvae	1.25, 2.5, 5 mg/L	7	[99]
	larvae	5 µg	7	[100]
Cd and terconazole	adult	Cd 0.01 mg/L tetraconazole: 0.1 mg/L	14	[101]
Chromium	larvae	1 mg/L Cr (III) and 1 mg/L Cr (VI)	7	[102]
Arsenic	larvae	[As^3+^] 10, 50, 100 ppb	20	[103]
	adult	[As^5+^] 10 μg/L	21	[104]
	larvae	[As^3+^] 50 ppb	60	[105]
Metal nanoparticles				
nTiO2; nZnO; nSe	larvae to adult	100 μg/L; 100 μg/L; 100 μg/L	90	[106]
nAg	larvae	0.25, 0.75, 1, 1.5, 2.5 mg/L	2	
	adult	33 and 100 μg/L	15, 45, 75	[107]

**Table 4:** Experimental conditions for microplastic exposure studies using zebrafish models: contaminant types, concentrations, particle sizes, and exposure durations.

Contaminant	Zebrafish Model	Concentration and MP Size	Days of Exposure	Ref.
Polystyrene	adult	1000 μg/L [0.5 and 50 μm diameter]	14	[136]
	adult	10 μg/L [sphere (15 um), fragment (15 um), and fiber (20 um)]	21	[137]
Polyethylene	larvae	20 mg/L [10–45 µm microspheres]	5 or 10	[138]
Polyethylene and polyester	adult	1 mg/L [1000 µm]	4	[139]
Polyethylene	adult	20 μg/L [70 nm and 13.5 µm]	21	[140]
Polypropylene	adult	50 mg/L [33.2 ± 14.43 µm]	14	[141]
Polystyrene	adult	10, 100 and 1000 [1 µm]	21	[142]
Polyethylene	adult	0.4 mg/L	15	[143]
Polyethylene; Polylactic	adult	25 000 particles/L [111.12 ± 30.53; 135.35 ± 37.12 µm]	15	[144]
Polyethylene; polyester	adult	200 µg/L and 1 mg/L [180 ± 210; 350 ± 220 µm]	30	[145]

**Table 5:** Contaminants and their effects on major phyla and genera of zebrafish gut microbiome.

	Phyla	Genera
Pesticides		
Methomyl	↑ Planctomycetes, Verrucomicrobiota, Actinobacteriota	↑ *Bacillus*, *Luteolibacter*
	↓ Fusobacteria	↓ *Shewanella*, *Pseudomonas*, *Gemmobacter*
LTC	↑ Proteobacteria, Firmicutes	No information
	↓ Fusobacteria	
CPZ	↑ Bacteroidetes, Firmicutes	↑ *Shewanella*, *Aeromonas*, *Chitinilyticum*, *Desulfovibrio*, *Paracoccus*, *Anaerobacillus*
Dieldrin	↑ Verrucomicrobiota	↑ *Defluviimonas*
	↓ Firmicutes, Clostridiales, Betaproteobacteria	↓ *Sphingomonas*, *Pseudomonas*
PTH	↑ Firmicutes	No information
Acetochlor	↑ Proteobacteria, Actinobacteriota	↑ *Gemmobacter*, *Xanthobacter*, *Bosea*, *Nocardia*, *Methylobacterium-Methylorubrum*
	↓ Fusobacteriota, Bacteroidota, Firmicutes	
IMI	↑ Fusobacteria	↑ *Cetobacterium*, *Paracoccus*, *Flavobacterium*, *Neisseriaceae*, *Acetobacteraceae*
	↓ Proteobacteria	↓ *Ralstonia*, *Bacillaceae*
Metamifop	No significant difference	↑ *Rhodobacter*, *Pelomonas*, *Ralstonia*
		↓ *Psychrobacter*, *Aeromonas*
Difenoconazole	↑ Firmicutes	↑ *Plesiomonas*, *Aeromonas*, *Ochrobactrum*, *Gemmobacter*, *Shewanella*, *Bacteroides*
		↓ *Cetobacterium*
IMZ	↓ Bacteroidetes, Proteobacteria	↑ *Rhodobacter*, *Shewanella*, *Bosea*, *Aeromonas*, * Acinetobacter*, *Mycoplasma*
		↓ *Bacteroides*
CYP	↑ Bacteroidota	↑ *Preplasmiviricota*, *Microsporidia*
	↓ Fusobacteriota	↓ *Alphaproteobacteria*
**Heavy metals**		
Lead	↑ Firmicutes, Bacteroidetes	↑ *p_Proteobacteria*, *c_Gammaproteobacteria*, *c_Alphaproteobacteria*
	↓ Proteobacteria, Fusobacteria	↓ *Prevotella*, *Corynebacterium*, *Ruminococcaceae_Ruminococcus*
Cadmium	↑ Firmicutes, Fusobacteria	↑ *Shewanella*, *Achromobacter*
	↓ Proteobacteria, Bacteroidetes	↓ *Xanthobacter*
Arsenic	↑ Fusobacteriota	↑ *Cetobacterium*
**Antibiotics and other medicines**		
FFC	↑ Verrucomicrobiota, Proteobacteria	↑ *Shinella*, *Reyranella*, *Bosea*
	↓ Bacteroidetes, Firmicutes	↓ *Vibrio*, *Flavobacterium*, *Mycobacterium*
SMX/TMP	↑ Proteobacteria	↓ *Cetobacterium somerae*
	↓ Fusobacteria	
Ery	↑ Proteobacteria	↑ *Aeromonas veronii*
	↓ Firmicutes	↓ *Cetobacterium somerae*
OTC	↑ Fusobacteria	
	↓ Firmicutes, Bacteroidetes, *Actinobacteria*, Verrucomicrobia	
Cortisone	↑ Firmicutes	↑ *Defluviimonas*
	↓ Bacteroidetes	
Norethindrone	↓ Bacteroidetes, Fusobacteria	↑ *Gordonia*, *Crenobacter*, *Bosea*
		↓ *Shewanella*
**Microplastics**		
Polystyrene	↑ Fusobacteria, Firmicutes	↑ *Cetobacterium*, *Plesiomonas*, *Aeromonas*, *Shewanella*, *Reyranella*, *Stenotrophomonas*, *Acidovorax*
	↓ Bacteroidetes	↓ *Exiguobacterium*, *Microbacterium*, *Methylobacterium*
Polyethylene	↑ Proteobacteria	↑ *Acinetobacter*
	↓ Tenericutes	↓ *Cerobacterium*
Polypropylene	No significant changes	↑ *Flavobacterium*, *Bacteroides*, *Rhodobacter*, *Stenotrophomonas*, *Ralstonia*, *Vogesella*, *Plesiomonas*
		↓ *Pseudomonas*
Polylactic	↑ Acidobacteria, Tenericutes	↑ *Bacteroides*, *Carnobacterium*, *Rhizobium*, *Gemmobacter*, *Cloacibacterium*
	↓ Fusobacteria	↓ *Xanthobacter*, *Ancylobacter*, *Luteolibacter*, *Azorhizobium*, *Lactobacillus*, *Methylobacterium*, *Cetobacterium*, *Aeromonas*, *Porphyromonadaceae*

## Data Availability

No new data were created or analyzed in this study. Data sharing is not applicable to this article.
